# Prevalence of human papillomavirus DNA sequences in an area with very high incidence of cervical carcinoma.

**DOI:** 10.1038/bjc.1994.375

**Published:** 1994-10

**Authors:** C. C. Pao, S. M. Kao, G. C. Tang, K. Lee, J. Si, S. Ruan

**Affiliations:** Department of Biochemistry, Chang Gung Medical College, Taipei, Taiwan, China.

## Abstract

**Images:**


Br. J. Cancer (1994), 70, 694-696 © Macmillan Press Ltd., 1994

### Prevalence of human papillomavirus DNA sequences in an area with very high incidence of cervical carcinoma

C.C. Pao[1], S.M. Kao[1], G.-C. Tang[1], K. Lee[2], J. Si[2] & S. Ruan[3]

[1]*Department of Biochemistry, Chang Gung Medical College, Taipei, Taiwan, China;* [2]*Department of Biophysics, Institute of Basic Medical Sciences, Chinese Academy of Medical Sciences, Beijing, China;* [2]*Institute of Cervical Cancer Research, LueYang County, Shanxi, China.*

**Summary** To improve our understanding of the relationship and possible associations between human papillomavirus (HPV) infection and the development of cervical malignancies, the presence of multiple types of HPV DNA sequences in cervical carcinoma was determined in Chinese citizens living in two different geographical locations where the incidences of cervical carcinoma are either relatively low or extremely high. HPV DNA sequences were found in 88.5% (54 of 61) of Chinese cervical carcinoma patients living in Taiwan, where the prevalence of cervical carcinoma is 23.7 per 100,000 women. In contrast, in LueYang in Shanxi province, an area with a very high prevalence of cervical carcinoma (1,026 per 100,000 women), only 57.1% (28 of 49) of Chinese cervical carcinoma patients were found to be infected with genital HPV. This result seems to suggest that either the presence of HPV may have different implications in different populations or HPV infection may not be the only factor that determines the development of cervical carcinoma, at least in certain geographical areas. Recently acquired transient or chronic persistent HPV infection may have a different outcome with regard to cervical carcinogenesis. Alternatively, other factors, such as host determinants, may play a role in the development of cervical carcinoma.

Certain types of human papillomaviruses (HPVs) have been implicated as one of the major risk factors in the development of malignancies of the uterine cervix in women (Howley, 1991). DNA-based analyses have consistently revealed a high prevalence of HPV 16 and 18 DNA sequences in cervical carcinomas (Gissmann, 1984; Brescia *et al.*, 1986; Pfister, 1987; Xiao *et al.*, 1988; Arends *et al.*, 1990). However, factors other than HPV infections, such as host factors, have also been suggested to play important roles in the carcinogenesis of cervical carcinoma (zur Hausen, 1991).

The prevalence of and mortality from cervical carcinomas among Chinese women living in Taiwan have been reported to be 23.7 and 6.15 per 100,000 women respectively (Reported Cancer Incidence, 1988). Although Chinese living in mainland China have a very low rate of venereal disease (Wegman *et al.*, 1973; Committee on Scholarly Communication with the People's Republic of China, 1981), central China has one of the highest rates of cervical carcinoma in the world (Scherer, 1983; Peto & Hausen, 1986). For example, the prevalence of cervical carcinoma in LueYang in the Shanxi province of China is 1,026 per 100,000 women based on an epidemiological survey of 12,980 women residents conducted in 1983 (Zhang *et al.*, 1986). The mortality resulting from cervical carcinoma in LueYang was 54.5 per 100,000 women (Zhang *et al.*, 1986). Both of these figures were the highest among all counties in China.

We report in the following text our examination and analysis of the presence of multiple types of HPV DNA sequences in cervical carcinoma tissues of Chinese patients living in Taiwan and LueYang, where the prevalence of cervical carcinoma is relatively low or extremely high.

### Materials and methods

### *Patients and cervical carcinoma tissues*

Cervical carcinoma tissue sections from 61 Chinese patients living in Taiwan (where the prevalence of cervical carcinoma is relatively low) and from 49 Chinese patients living in LueYang in the Shanxi province of China (where the prevalence of cervical carcinoma is extremely high) were the subjects of this investigation. These tissue sections were prepared from either fresh-frozen or paraffin blocks with disposable blades to avoid any cross-contamination of HPV.

### *Determination of the presence of HPV DNA sequences*

Two consecutive 5 μm sections were cut from each of the paraffin blocks. One section was used for histopathological evaluation after staining (haematoxylin and eosin) and one for the extraction of total DNA with phenol and chloroform. The DNA was further purified by alcohol precipitation and then used for the determination of the presence of HPV DNA sequences by *in vitro* DNA amplification with consensus primers MY09 and MY11 purchased from Perkin-Elmer Cetus (Norwalk, CT, USA). These primers are capable of amplifying DNA from L1 ORF of genital HPV types 6, 11, 16, 18, 30, 31, 33, 35, 39, 40, 42, 43, 45, 51, 52, 53, 54, 55, 57, 58, 59 and from at least another 20 others as yet undetermined HPV types (Bauer *et al.*, 1991). Dermal HPV types 1, 5, 8, 26, 27, 41, 47 and 48 are also amplified with these primers. The sequences of the oligonucleotide primers were CGTCCMARRGAWACTGATC and GCMCAGGWCATAAYAATGG (where M = A + C, R = A + G, W = A + T, Y = C + T). DNA samples, deoxyribonucleoside triphosphates and primers were heated in buffer to 95°C for 5 min before *Taq* DNA polymerase (Perkin-Elmer Cetus) was added to the reaction mixture and reaction started in a thermocycler (Model 480, Perkin-Elmer Cetus). The temperatures of the reaction mixture were cycled 35 times through 95°C denaturation (30 s), 55°C annealing (30 s) and 72°C extension (1 min) with a 10 min incubation at 72°C at the end. Positive and negative control DNAs were always included in every polymerase chain reaction (PCR) assay. Portions of the amplified reaction mixture were separated by electrophoresis in 2% agarose gel, with pGEM-3 DNAs digested with a mixture of restriction endonucleases (*Hinf*I, *Rsa*I and *Sin*I) serving as size standards. HPV positivity was determined by the presence of 450 bp amplified HPV DNA by visual inspection under ultraviolet light after staining with ethidium bromide. Typing of HPV DNA was achieved by restriction endonuclease digestion (Lungu *et al.*, 1992) and by hybridisation to type-specific internal oligonucleotide (Pao *et al.*, 1991*a*, *b*). Adequacy and suitability of DNA for the amplification reactions were ensured by the amplification of a

Correspondence: C.C. Pao, Department of Biochemistry, Chang Gung Medical College, 259 Wen Hwa Road, KweiShan, TaoYuan, Taiwan 33332, China.
Received 10 August 1993; and in revised form 4 March 1994.

1,455 bp portion of the human β-globin gene DNA (Pao *et al.*, 1993).

*Precautions against contamination and false-positive results in PCR*

Because of the immense sensitivity of the PCR, every precaution was taken to minimise the possibility of contamination during sampling and subsequent processing (Pao *et al.*, 1991*a*, *b*). Steps were also taken to minimise sample-to-sample contamination and PCR product carry-over in order to avoid false-positive results. These precautions include aliquoting of all reagents, physical separation of pre- and post-PCR reactions and use of disposable containers whenever possible. Furthermore, all reagents were irradiated with ultraviolet light to inactivate any contaminated double-stranded DNA before sample DNA was added and the PCR started. Fifty nanograms of human DNA or 1 ng of *E. coli* DNA was used as a negative control, which always yielded negative results. Multiple reagent controls were also included in each PCR assay and gave negative results. Repeated DNA amplification assays of the same specimens at different times produced the same results.

### Results

The determination of the presence of HPV DNA sequences by PCR with consensus primers MY09 and MY11 is illustrated in Figure 1. The sensitivity of this method of detecting HPV types 16 and 18 DNA was determined to be less than 200 HPV genome equivalents based on amplification of either serial dilutions of purified cloned HPV DNA of known concentrations or of DNA prepared from CaSki and HeLa cells (data not shown). The adequacy of DNA available for amplification and the fact that DNA prepared from specimens obtained from Taiwan and LueYang were equally efficient for amplification were ensured by the ability to amplify a 1,455 bp portion of human β-globin gene (data not shown).

DNA sequences homologous to that of HPV were found in cervical carcinoma tissues of Chinese patients living both in Taiwan and in LueYang of Shanxi province (Table I). The HPV DNA-positive rates were 88.5% (54 of 61) and 57.1% (28 of 49) among these two groups of Chinese cervical cancer patients respectively. Forty-six of 54 of the HPV-positive specimens from Taiwan and 21 of 28 HPV-positive specimens from LueYang contained either type 16 or 18 DNA.

No visible correlation can be found between the HPV positivity and either the age of the patients at the time of diagnosis or the pathology and stages of the cervical carcinomas (data not shown).

### Discussion

HPV DNA sequences, predominantly the high-risk types of 16 and 18, were found very frequently in cervical carcinomas. Depending on the methods of detection used, HPV 16 and 18 DNA sequences were found in up to 90% of cervical carcinomas (Gissman, 1984; Brescia *et al.*, 1986; Pfister, 1987; Xiao *et al.*, 1988; Arends *et al.*, 1990; Lorincz *et al.*, 1992). Furthermore, immortalisation of primary genital epithelial cells *in vitro* by HPV 16 and 18 requires the expression of E6 and E7 oncoproteins of these viruses (Hawley-Nelson *et al.*, 1989). Some oncoprotein genes are also retained and expressed in cervical carcinomas and carcinoma-derived cell lines (Schneider-Gadicke & Schwarz, 1986; Smotkin & Wettstein, 1986). Despite all these reports, definitive proof of a causal role for HPV in cervical carcinoma is currently lacking. There is still controversy regarding the role of HPV in cervical malignancies. The small number of cervical carcinomas negative for HPV DNA may be genuinely unrelated to HPV infection or may contain other yet undetermined

**Figure 1** Agarose gel electrophoresis of amplified HPV DNA using consensus primers and the conditions described in the Materials and methods section. pGEM-3 DNA digested with a mixture of restriction endonucleases (*Hinf*I, *Rsa*I and *Sin*I) was used as size standards in lanes A and J, and the sizes are (from top to bottom) 2,645, 1,605, 1,198, 676, 517, 460, 396, 350, 222, 179, 126, 75, 65, 51 and 36 bp. Lanes B and C are the amplification of DNA from purified cloned HPV types 6 and 11 DNA respectively. Lanes D (HPV positive) and E (HPV negative) are the amplification of DNA from cervical cancer tissue sections from two patients. Lane F is the amplification of DNA from finger-like papillomatosis lesions from one patient (HPV negative). Lane G is the amplification of 50 ng of human DNA with the HPV consensus primers MY09 and MY11. Lanes H and I are the amplification of CaSki and HeLa cells, respectively, which are known to contain HPV types 16 and 18 respectively.

**Table I** HPV in Chinese cervical carcinoma patients living in Taiwan and in LeuYang in the Shanxi province in China

| *HPV* | *Taiwan* | *LeuYang* |
|---|---|---|
| Positive | 54 | 28 |
| Negative | 7 | 21 |
| Total | 61 | 49 |
| Per cent positive | 88.5 | 57.1 |

HPV, or low copy numbers of viral DNA which, so far, have proven difficult to detect. On the other hand, because of the use of extremely sensitive PCR, HPV DNA has been found in a substantial percentage of cervical smears of women without cytological evidence of abnormalities (Young *et al.*, 1989; Nakazawa *et al.*, 1992). The role these so-called 'subclinical' HPV may play, if any, in the development of cervical malignancies is unclear.

The reasons for the relatively low HPV prevalence in LueYang is not known at the present time, but it is not completely surprising because almost all of the residents in LueYang live all their lives in conservative, farming communities with puritanical mores. This low HPV prevalence in cervical carcinomas from LueYang cannot be explained by the use of different detection methods because all of our specimens were analysed in the same laboratory using the same primers and amplification conditions. Low HPV prevalence in cervical carcinomas in LueYang may be clinically important because 90% of cervical carcinomas from Chengdu in Sichuan province, which is not too far from LueYang, were found to contain DNA related to HPV 16 and or 18 (Xiao *et al.*, 1988). It has to be stated, though, that we cannot completely rule out the possibility that HPV other than the more than 40 types that could be detected by the primers we used might be responsible for, or involved in, the carcinogenesis of cervical carcinomas in LueYang. However, if this is the case, then this putative HPV would have to infect as frequently as, if not more frequently than, HPV 16 and 18 did in cervical carcinomas reported elsewhere. So far, no HPV has been found in cervical carcinomas more frequently than types 16 and 18. Kjaer *et al.* (1993) recently reported that, using both DNA hybridisation and PCR methods, they could find no significant difference in HPV prevalence between cervical carcinoma patients from high-risk and low-risk areas.

There is presently no explanation for the relatively low HPV prevalence in a population with extremely high cervical carcinoma incidence in China. Host factors are among the possible causes that might have contributed to this extremely high incidence of cervical carcinoma in LueYang. It is not impossible that these residents are genetically predisposed to be at-risk for cervical carcinoma owing to mutational alterations in certain host genes that possess tumour-suppressive activities. This notion is in accordance with the fact that LueYang is an isolated inland farming community and local residents very frequently marry residents from within the same community. Studies are also currently under way to determine whether transient recently acquired versus chronic persistent HPV infections may exist in these two patient populations which may have a different outcome with regard to the development of cervical malignancies.

Whatever the underlying reasons for the extremely high cervical carcinoma incidence in LueYang, our results seem to suggest that HPV may not be the only factor that determines the development of cervical carcinoma, at least in certain geographical areas. Cervical carcinomas in these patients may provide us with an excellent opportunity to examine the possible role of host factors, including tumour-suppressor genes, in the carcinogenesis of cervical carcinoma.

This study was supported by Research Grant NSC81-0412-B182-028 from the National Science Council of Republic of China and Medical Research Grant CMRP-343 from Chang Gung Medical College and Memorial Hospital, both awarded to C.C.P. The authors acknowledge the encouragement and support of Dr Delon Wu and Dr Chau-Hsiung Chang. The assistance of Dr Chyong-Huey Lai and Dr Horng-Chyi Chang of the Department of Obstetrics and Gynecology of Chang Gung Memorial Hospital is also appreciated.

### References

ARENDS, M.J., WYLLIE, A.H. & BIRD, C.C. (1990). Papillomaviruses and human cancer. *Human Pathol.*, **21**, 686–698.

BAUER, H.M., TING, Y., GREER, C.E., CHAMBERS, J.C., TASHIRO, C.J., CHIMERA, J., REINGOLD, A. & MANOS, M.M. (1991). Genital human papillomavirus infection in female university students as determined by a PCR-based method. *JAMA*, **265**, 472–477.

BRESCIA, R.J., JENSON, A.B., LANCASTER, W.D. & KURMAN, R.J. (1986). The role of human papillomaviruses in the pathogenesis and histologic classification of precancerous lesions of the cervix. *Hum. Pathol.*, **17**, 552–559.

COMMITTEE ON SCHOLARLY COMMUNICATION WITH THE PEOPLE'S REPUBLIC OF CHINA (1981). *Rural Health in the People's Republic of China*, p. 15. Department of Health and Human Services: Washington, DC.

GISSMANN, L. (1984). Papillomaviruses and their association with cancer in animals and in man. *Cancer Surv.*, **3**, 161–181.

HAWLEY-NELSON, P., VOUSDEN, K.H., HUBBERT, N.L., LOWY, R. & SCHILLER, J.T. (1989). HPV 16 E6 and E7 proteins co-operate to immortalize human foreskin keratinocytes. *EMBO J.*, **8**, 3905–3910.

HOWLEY, P. (1991). Role of the human papillomaviruses in human cancer. *Cancer Res.*, **51** (Suppl.), 5019s–5022s.

KJAER, S.K., DE CILLIERS, E.M., CAGLAYAN, H., SVARE, E., HAUGAARD, B.J., ENGHOLM, G., CHRISTENSEN, R.B., MOLLER, K.A., POLL, P. & JENSEN, H. (1993). Human papillomavirus, herpes simplex virus and other potential risk factors for cervical cancer in a high-risk area (Grenland) and a low-risk area (Denmark) – a second look. *Br. J. Cancer*, **67**, 830–837.

LORINCZ, A.T., REID, R., JENSON, B., GREENBERG, M.D., LANCASTER, W. & KURMAN, R.J. (1992). Human papillomavirus infection of the cervix: relative risk associations of 15 common anogenital types. *Obstet. Gynecol.*, **79**, 328–337.

LUNGU, O., WRIGHT, Jr, T.C. & SILVERSTEIN, S. (1992). Typing of human papillomaviruses by polymerase chain reaction amplification with L1 consensus primers and RFLP analysis. *Mol. Cell. Probes*, **6**, 145–152.

NAKAZAWA, A., INOUE, M., SAITO, J., SASAGAWA, T., UEDA, G. & TANIZAWA, O. (1992). Detection of human papillomavirus types 16 and 18 in the exfoliated cervical cells using the polymerase chain reaction. *Int. J. Gynecol. Obstet.*, **37**, 13–18.

PAO, C.C., LIN, C.Y., CHANG, Y.L., TSENG, C.J. & HSUEH, S. (1991*a*). Human papillomaviruses and small cell carcinoma of the uterine cervix. *Gynecol Oncol.*, **43**, 206–210.

PAO, C.C., LIN, S.S., LIN, C.Y., MAA, J.S., LAI, C.H. & HSIEH, T.T. (1991*b*). Identification of human papillomavirus in peripheral blood mononuclear cells by DNA amplification method. *Am. J. Clin. Pathol.*, **95**, 540–546.

PAO, C.C., LIN, C.Y., TANG, G.C., SUN, C.F. & HSIEH, T.T. (1993). Detection of β-thalassemia carrier by direct analysis of β-globin gene lesions. *Biochem. Biophys. Res. Commun.*, **191**, 1118–1123.

PETO, R. & ZUR HAUSEN, H. (eds) (1986). *Viral Etiology of Cervical Cancer*. Cold Spring Harbor Laboratory Press: Cold Spring Harbor, NY.

PFISTER, H. (1987). Human papillomaviruses and genital cancer. *Adv. Cancer Res.*, **48**, 113–147.

REPORTED CANCER INCIDENCE (1988). In *Health Statistics*, Vol. 1, *General Health Statistics*, pp. 49–49. Department of Health: Taipei, Republic of China.

SCHERER, J.L. (ed.) (1983). *China Facts and Figures* (1983), p. 408. Academic International Press: New York.

SCHNEIDER-GADICKE, A. & SCHWARZ, E. (1986). Different human cervical carcinoma cell lines show similar transcription pattern of human papillomavirus type 18 early genes. *EMBO J.*, **5**, 2285–2292.

SMOTKIN, D. & WETTSTEIN, F.O. (1986). Transcription of human papillomavirus type 16 early genes in a cervical cancer and a cancer-derived cell line and identification of the E7 protein. *Proc. Natl Acad. Sci. USA*, **83**, 4680–4684.

WEGMAN, M.E., LIN, T.Y. & PURCELL, E.F. (eds) (1973). *Public Health in the People's Republic of China*, p. 176. Josiah Macy, Jr. Foundation: New York.

XIAO, X., CAO, M., MILLER, T.R., CAO, Z.Y. & YEN, T.S.B. (1988). Papillomavirus DNA in cervical carcinoma specimens from central china. *Lancet*, **ii**, 902.

YOUNG, L.S., BEVAN, I.S., JOHNSON, M.A., BLOMFIELD, P.I., BROMIDGE, T., MALTLAND, N.J. & WOODMAN, C.B.J. (1989). The polymerase chain reaction: a new epidemiological tool for investigating cervical human papillomavirus infection. *Br. Med. J.*, **298**, 14–18.

ZHANG, J.M., RUAN, S.X., LIU, D.K., KUN, H.S., LEE, B.L., BIEN, S.Y. & ZHANG, R.T. (1986). The distribution and characteristics of carcinoma of cervix uteri in LueYang county. *Chin. J. Epidemiol.*, **7**, 343–345.

ZUR HAUSEN, H. (1991). Viruses in human cancers. *Science*, **254**, 1167–1173.


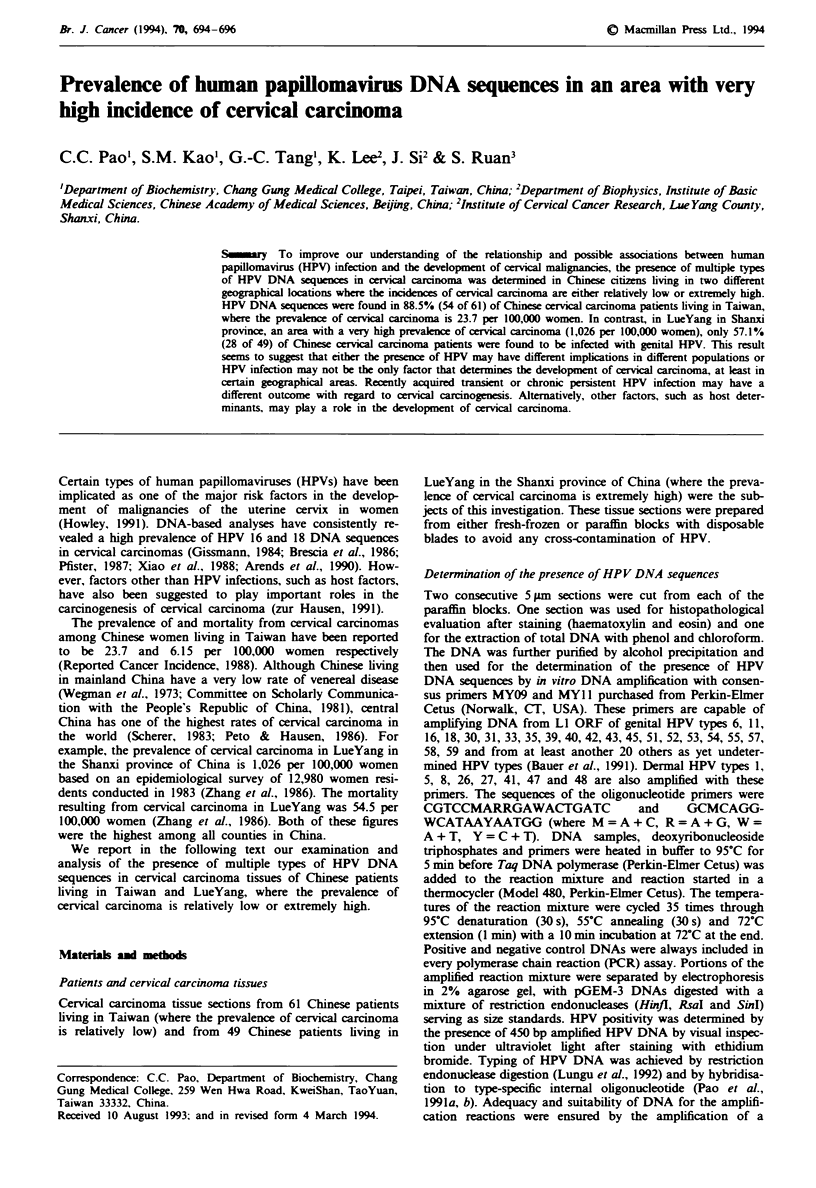

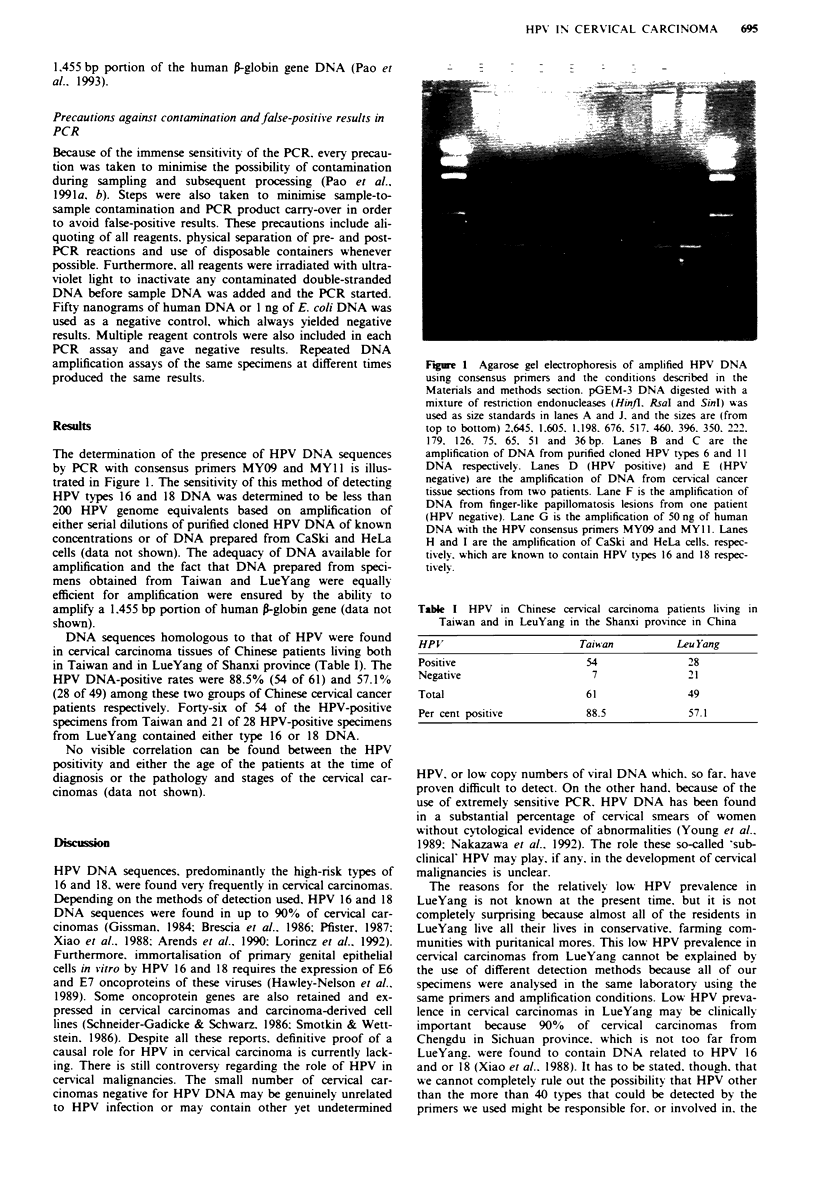

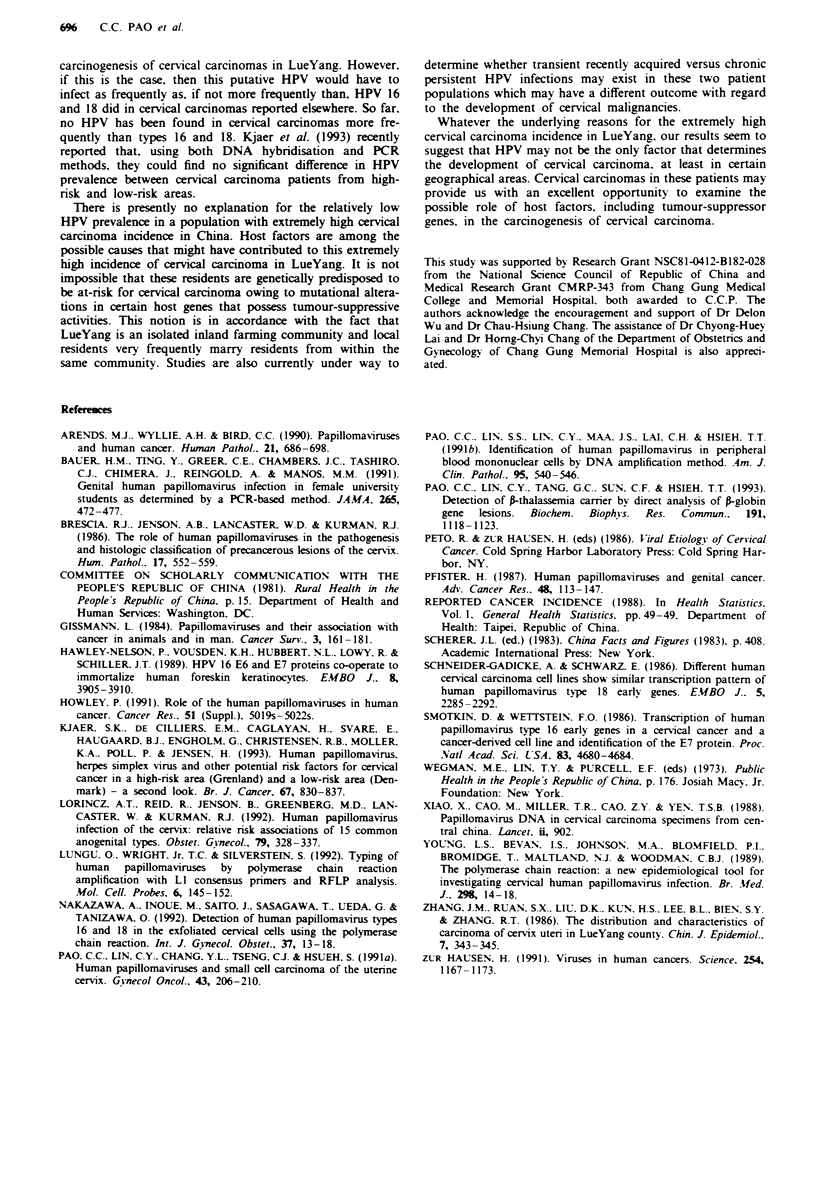


## References

[OCR_00314] Arends M. J., Wyllie A. H., Bird C. C. (1990). Papillomaviruses and human cancer.. Hum Pathol.

[OCR_00319] Bauer H. M., Ting Y., Greer C. E., Chambers J. C., Tashiro C. J., Chimera J., Reingold A., Manos M. M. (1991). Genital human papillomavirus infection in female university students as determined by a PCR-based method.. JAMA.

[OCR_00323] Brescia R. J., Jenson A. B., Lancaster W. D., Kurman R. J. (1986). The role of human papillomaviruses in the pathogenesis and histologic classification of precancerous lesions of the cervix.. Hum Pathol.

[OCR_00341] Hawley-Nelson P., Vousden K. H., Hubbert N. L., Lowy D. R., Schiller J. T. (1989). HPV16 E6 and E7 proteins cooperate to immortalize human foreskin keratinocytes.. EMBO J.

[OCR_00347] Howley P. M. (1991). Role of the human papillomaviruses in human cancer.. Cancer Res.

[OCR_00349] Kjaer S. K., de Villiers E. M., Cağlayan H., Svare E., Haugaard B. J., Engholm G., Christensen R. B., Møller K. A., Poll P., Jensen H. (1993). Human papillomavirus, herpes simplex virus and other potential risk factors for cervical cancer in a high-risk area (Greenland) and a low-risk area (Denmark)--a second look.. Br J Cancer.

[OCR_00359] Lorincz A. T., Reid R., Jenson A. B., Greenberg M. D., Lancaster W., Kurman R. J. (1992). Human papillomavirus infection of the cervix: relative risk associations of 15 common anogenital types.. Obstet Gynecol.

[OCR_00365] Lungu O., Wright T. C., Silverstein S. (1992). Typing of human papillomaviruses by polymerase chain reaction amplification with L1 consensus primers and RFLP analysis.. Mol Cell Probes.

[OCR_00372] Nakazawa A., Inoue M., Saito J., Sasagawa T., Ueda G., Tanizawa O. (1992). Detection of human papillomavirus types 16 and 18 in the exfoliated cervical cells using the polymerase chain reaction.. Int J Gynaecol Obstet.

[OCR_00377] Pao C. C., Lin C. Y., Chang Y. L., Tseng C. J., Hsueh S. (1991). Human papillomaviruses and small cell carcinoma of the uterine cervix.. Gynecol Oncol.

[OCR_00388] Pao C. C., Lin C. Y., Tang G. C., Sun C. F., Hsieh T. T. (1993). Detection of beta-thalassemia carrier by direct analysis of beta-globin gene lesions.. Biochem Biophys Res Commun.

[OCR_00382] Pao C. C., Lin S. S., Lin C. Y., Maa J. S., Lai C. H., Hsieh T. T. (1991). Identification of human papillomavirus DNA sequences in peripheral blood mononuclear cells.. Am J Clin Pathol.

[OCR_00397] Pfister H. (1987). Human papillomaviruses and genital cancer.. Adv Cancer Res.

[OCR_00410] Schneider-Gädicke A., Schwarz E. (1986). Different human cervical carcinoma cell lines show similar transcription patterns of human papillomavirus type 18 early genes.. EMBO J.

[OCR_00416] Smotkin D., Wettstein F. O. (1986). Transcription of human papillomavirus type 16 early genes in a cervical cancer and a cancer-derived cell line and identification of the E7 protein.. Proc Natl Acad Sci U S A.

[OCR_00429] Xiao X., Cao M., Miller T. R., Cao Z. Y., Yen T. S. (1988). Papillomavirus DNA in cervical carcinoma specimens from central China.. Lancet.

[OCR_00432] Young L. S., Bevan I. S., Johnson M. A., Blomfield P. I., Bromidge T., Maitland N. J., Woodman C. B. (1989). The polymerase chain reaction: a new epidemiological tool for investigating cervical human papillomavirus infection.. BMJ.

[OCR_00445] zur Hausen H. (1991). Viruses in human cancers.. Science.

